# The interplay between programmed death ligand 1 (PD-L1) expression and human papillomavirus (HPV) genotypes in cervical carcinomas: findings of a Nigerian Tertiary Hospital

**DOI:** 10.11604/pamj.2024.48.90.42773

**Published:** 2024-07-07

**Authors:** Sebastian Anebuokhae Omenai, Mustapha Akanji Ajani

**Affiliations:** 1 Department of Anatomical Pathology, Edo State University, Uzairue, Edo State, Nigeria,; 2 Department of Pathology, University College Hospital, Ibadan, Oyo State, Nigeria

**Keywords:** Uterine cervical neoplasm, human papillomavirus 35, B7-H1 antigen, immune checkpoint inhibitors, real-time polymerase chain reactions

## Abstract

**Introduction:**

cervical cancer is primarily driven by high-risk human papillomavirus infections. It is a leading cause of cancer-related deaths among women globally. The emergence of immunotherapeutic approaches, particularly programmed death ligand-1 (PD-L1) inhibitors, has shown promise in various cancers. This study aims to investigate the correlation between PD-L1 expression and HPV status in cervical carcinoma samples from a Nigerian tertiary hospital.

**Methods:**

the study was conducted in the Department of Pathology of our hospital. The study materials were 101 cases of archival formalin-fixed paraffin-embedded (FFPE) tissue blocks that met the study criteria recruited retrospectively from January 2012 to December 2016. Immunohistochemistry for PD-L1 was done and real time PCR for HPV DNA was done using CFX96. The data were then analyzed using SPSS version 23. P < 0.05 was considered significant.

**Results:**

high-risk HPV detection rate was 51%. The two most common genotypes were HPV 16 (84.3%) and HPV 35 (17.6%). The predominant infections were single genotypes occurring in 80.4% of the cases. There was no correlation of HPV status with PD-L1, histological grade or type of cervical carcinoma. High-risk HPV did not show any distribution pattern with age groups of patients.

**Conclusion:**

human papillomavirus 16 is the most predominant cause of cervical carcinoma. There are some environmental variations in the frequency of other genotypes with HPV 35 being relatively more common than HPV 18 in this study. Programmed death ligand-1 was positive in 47% of the cases but did not show any correlation with the HPV infection status.

## Introduction

Cervical cancer is a significant health concern in most countries especially developing countries such as Nigeria. It is the most common gynaecological malignancy in Nigeria and sub-Saharan Africa [1,2]. Although, globally it is the fourth most common cancer in females, it is the second most common female malignancy in Nigeria after breast cancer [3,4]. The aetiology of cervical cancer is attributable to persistent infection with high-risk human papillomavirus (HPV) [5,6]. The most common high-risk HPV include types 16, 18, 31, 33, 35, 45, 52, 58 [7-9]. Types 16 and 18 have the highest prevalence globally [10]. There is some regional variation in the prevalence of some genotypes, some reports from West Africa and South America have reported low numbers for HPV type 18 [11-13]. Notably, high-risk HPV genotypes exhibit a propensity for persistent infections, a critical factor in the subsequent development of malignancies [6]. Persistence often leads to the integration of HPV deoxyribonucleic acid (DNA) into the host genome [6]. This integration disrupts the delicate balance of the cell cycle control mechanisms, tipping the scales towards uncontrolled cell division-a hallmark of cancer progression. E6 and E7 oncoproteins are central to HPV-induced oncogenesis [6]. E6, by binding to and degrading the tumour suppressor protein p53, circumvents cellular defences against DNA damage. Meanwhile, E7's interaction with the retinoblastoma (Rb) protein promotes unbridled cell proliferation, creating a conducive environment for malignant transformation. The E6 and E7 oncoproteins synergize to dysregulate the cell cycle, resulting in the accumulation of genetic mutations and genomic instability [6]. This chaotic cellular environment provides fertile ground for the transformation of infected cells into cancerous entities.

Human papillomavirus has evolved mechanisms to evade the host immune response, allowing infected cells to persist. This immune evasion not only facilitates the prolonged survival of infected cells but also contributes to the progression of HPV-associated lesions to invasive cancer [6,14,15]. Programmed death-ligand 1 (PD-L1) has emerged as a key orchestrator of immune evasion in the tumour microenvironment. E5 oncoprotein of HPV activates EGFR which then leads to increase expression of PD-L1 with subsequent apoptosis of T-cells [6,16]. In normal physiological settings, the PD-L1/PD-1 interaction serves as a crucial mechanism for immune regulation [16]. By binding to PD-1, PD-L1 sends inhibitory signals to T cells, preventing them from mounting an overly aggressive immune response and maintaining immune homeostasis [16]. In cancer, PD-L1 expression on tumour cells engages with the PD-1 receptor on T cells, initiating inhibitory signals that exhaust and impairs the immune response. This molecular interaction creates an immune-privileged niche in the tumour microenvironment, enabling uncontrolled tumour growth [16,17]. The clinical implications are significant, leading to the development of immunotherapies targeting PD-L1/PD-1 interactions. These therapies aim to disrupt immune checkpoints, reinvigorating the immune response against cancer [16]. Human papillomavirus (HPV)-induced immune responses and the subsequent upregulation of PD-L1 by cancer cells contribute to an immunosuppressive microenvironment, allowing for immune evasion [6]. In this study, we determined the frequency of HPV-associated cervical cancer by HPV DNA analysis, as well as the expression of PD-L1 by these cancers via immunohistochemistry. This study aims to explore the intricate connection between PD-L1 and HPV status in cervical carcinomas, offering valuable insights into cervical carcinoma in Nigerian patients.

## Methods

**Study design and materials:** this is a retrospective study of all formalin fixed paraffin embedded (FFPE) blocks of cervical cancer cases diagnosed in the Department of Pathology over a five-year period spanning January 2012 to December 2016. Histopathological records and relevant biodata and clinical information were retrieved from the Departmental records. All histologically diagnosed cases of cervical carcinoma in the departmental records during the study period were included in the study. Some cases had missing FFPE blocks, or insufficient tumour for immunohistochemistry and DNA extraction for molecular analysis by polymerase chain reaction. These cases were then excluded from the study.

**Programmed death ligand-1 immunohistochemistry:** immunohistochemistry was done following the manufacturer´s protocol. Polyclonal antibody for PD-L1 is anti PD-L1 GTX104763 rabbit antibody (Genetex, Taiwan) used at 1: 500 dilutions. The slides were incubated with the primary antibody, rabbit anti-PD-L1 antibody in a humidified chamber at room temperature for one hour, and then incubated for 15 minutes with MACH 4TM mouse probe (Biocare medicals) at room temperature. MACH 4TM HRP-polymer (horseradish peroxidase polymer) (Biocare medicals) was added to the slides and allowed to incubate for 15 minutes at room temperature, and then washed using wash buffer. The DAB (3,3 diaminobenzidine) (Biocare medicals) chromogen substrate was added next and allowed to incubate for seven minutes. The slides were counterstained with Haematoxylin for ten seconds at room temperature [18]. The antibody is visualized as membrane staining [18]. The combined positive score (CPS) was used in assessing the PD-L1 expression. CPS counts the number of positive malignant cells (showing complete and/or partial circumferential linear plasma membrane staining), lymphocytes and macrophages divided by the overall viable tumour cell number multiplied by 100 [19]. Cervical cancers were regarded as expressing PD-L1 positivity if CPS is ≤1%. Negative PD-L1 expression is regarded as <1%.

**Human papillomavirus genotyping:** deoxyribonucleic acid extraction was done using Zymo Quick DNA FFPE Extraction Kit [Zymoresearch USA]. Tissues were sectioned using a microtome and 260μL Proteinase K storage buffer was added to reconstitute lyophilized proteinase K at 20mg/ml and then vortexed to dissolve and stored at -20°C. Genomic DNA wash 2 concentrate and RNase A were also reconstituted according to the manufacturer [Zymoresearch USA]. Two hundred and fifty (250) μL Beta- mercaptoethanol was added to the Genomic lysis buffer (50ml) for optimal performance. The FFPE sectioned ribbons were deparaffinized by adding 400 μL of deparaffinization solution to the sample; and incubated at 55°C for 20mins, and then vortexed briefly. The sample was then digested and the DNA then passed through a purification process. The spectrophotometric quantification method was adopted for DNA quantification, using an instrument known as the Nanodrop. It is designed to measure the absorbance and calculate the concentration of nucleic acids (260nm) and purified proteins (280nm). This would include double-stranded DNA (dsDNA), single-stranded DNA (ssDNA), RNA and purified proteins. The ratio of the absorbance at 260 and 280nm (A260/280) is used to assess the purity of nucleic acids. For pure DNA, A260/280 range between 1.8-2.0. The human papillomavirus (HPV) genotyping was carried out using the Seegene Anyplex HPV 28 Detection kit [Seegene, Seoul, South Korea]. The Kit is composed of: 4X HPV 28 A TOM (Primer A), 4X HPV 28 B TOM (Primer B), EM1 (Master Mix)- 2 vials, RNase- free water- 2 vials, three positive control tubes (PC1, PC2, PC3). Primer A can detect 14 types of high risk and Internal Controls which include: HPV 66, 45, 58, 51, 59, 16, 33, 39, 52, 35, 18, 56, 68, 31; while Primer B can detect 5 types of high risk, which include: 26, 69, 73, 82, 53, and low 9 types of low risk 43, 54, 70, 61, 6, 44, 40, 11, 42. Each positive control includes clones for 5 targets in A set, and 5 targets in B set. To run the positive control reaction, three PCR tubes were prepared for each set, making six PCR tubes in total (for set A and B). The dyes used in the Seegene Anyplex HPV 28 detection kit are five in number, and they include: FAM (Fluorescein amidite), HEX (Hexachloro-fluorescein), Cal Red 610, Quasar 670, and Quasar 705. The experimental set up used for the genotyping is the real time polymerase chain reaction (qPCR) platform. Specifically, the Bio-Rad CFX 96 qPCR platform was the adopted platform. The data was exported from the assay and it was run with the Seegene viewer app [Seegene, Seoul, South Korea].

**Data analysis:** the obtained data were stored into excel spreadsheet (Microsoft, Redmond, Washington, The United States of America) and then exported to IBM SPSS statistics (version 23 IBM Corporation, Armonk, New York) for statistical analysis. Descriptive statistics was used to summarize HPV detection status, PD-L1 immunohistochemical expression and HPV genotypes and recorded as frequencies and percentages. The Chi-square test was used to test for a relationship between PD-L1 status and the HPV status and genotypes. The level of significance was set as p < 0.05.

**Ethical clearance:** it was obtained from the Joint Ethical Review Committee of the College of Medicine, University of Ibadan and the University College Hospital, Ibadan. The study was conducted in compliance with the Helsinki declaration of ensuring confidentiality and dignity of patients. The study didn´t involve patients directly and all archival materials used were treated as anonymous. Data collected were stored in a laptop that was secured by a password.

## Results

**Figure 1:**
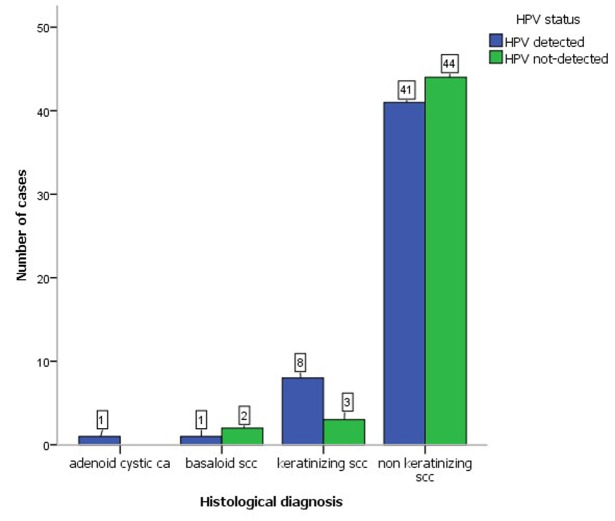
clustered bar charts showing high-risk human papilloma virus detection status within histological types of cervical cancer; (SCC: squamous cell carcinoma, ca: carcinoma)

The review period had a total number of 276 cases of cervical cancer diagnosed. After exclusion of cases with missing FFPE blocks and those with insufficient materials for quality DNA extraction we had only 101 cases representing 36.6% of the cervical cancer cases available for genotyping. The age of the patients at diagnosis ranged from 25 years to 83 years with a mean age of 57 years. Majority of the patients (70.1%) were between 30 and 50 years at the time of diagnosis. High-risk HPV was detected in 51% of the cases. The most prevalent HPV genotype was HPV 16 accounting for 84.3% followed distantly by HPV 35 with 17.6% (Table 1). Single infections (80.4%) were more common than multiple infections (Table 1). The most frequent multiple infections observed was co-infection with HPV 16 and 18, which was present in 4 cases (7.8%). There were two cases (3.9%) of co-infection with HPV 16 and 56. There were two cases of triple infections; HPV 16, 35, 58 and HPV 16, 35, 66. The histological grade of cervical cancers did not show any correlation with HPV status (χ^2^(2) =1.989, p = 0.370). There was no correlation between HPV status and the age group of patients at diagnosis (Table 2). There was no association between the histological types and HPV detection status (Figure 1). Programmed death ligand-1 was positive in 47% of the cases and there was no correlation between the PD-L1 status and the detection of HPV (χ^2^(1) = 0.623, p = 0.370) nor with the genotypes detected (χ^2^(9) = 11.529, p = 0.241) (Table 3).

**Table 1:** frequency distribution of high-risk HPV and PD-L1 in cervical carcinoma

S/N	Variable	Frequency	%
**A**	**Age distribution**		
	<30 years	1	01%
	30-49 years	28	28%
	≥50 years	71	71%
**B**	**Numbers of HPV genotype per case**		
	Single infection	41	80.4%
	Double infections	8	15.7%
	Triple infections	2	3.9%
**C**	**PD-L1 status**		
	Positive	47	47%
	Negative	53	53%
**D**	**HPV status**		
	Hr HPV detected	51	51%
	Hr HPV not detected	49	49%
**E**	**HPV genotypes**		
	HPV 16	43	84.3%
	HPV 18	4	7.8%
	HPV 31	2	3.9%
	HPV 35	9	17.6%
	HPV 56	2	3.9%
	HPV 58	1	2.0%
	HPV 66	2	3.9%

Hr HPV: high risk human papilloma virus

**Table 2:** relationship of high-risk HPV and pathological characteristics of cervical cancer

Variable	HPV status (%)		X^2^	P-value
	**Negative**	**Positive**		
**Histological grade**			1.989	0.370
Well differentiated	7 (41.2)	10 (58.8)		
Moderately differentiated	29 (55.8)	23 (44.2)		
Poorly differentiated	13 (41.9)	18 (58.1)		
**Histological type**			3.673	0.299
Adenoid cystic carcinoma	0 (0.0%)	1 (100%)		
Basaloid SCC	2 (66.7%)	1 (33.3%)		
Keratinizing SCC	3 (27.3%)	8 (72.7%)		
Non keratinizing SCC	44 (51.8%)	41 (48.2%)		
**Age group**			0.813	0.367
Less than 30 yrs	1 (100%)	0(0%)		
30 - 49 yrs	12 (42.9%)	16 (57.1%)		
50 years and above	36 (50.7%)	35 (49.3%)		
**PD-L1 status**			0.623	0.370
Positive	25 (53.2%)	22 (46.8%)		
Negative	24 (45.3%)	29 (54.7%)		

HPV: human papilloma virus; SCC: squamous cell carcinoma; PD: programmed death-ligand

**Table 3:** PD-L1 expression in relation to different high-risk HPV genotypes

	PD-L1 status	
**HPV genotypes**	**Negative n (%)**	**Positive n (%)**
HPV 16	18 (54.5)	15 (45.5)
HPV 16, 35 and 58	1 (100)	0 (0)
HPV 16, 35 and 66	0 (0)	1 (100)
HPV 16 and 18	4 (100)	0 (0)
HPV 16 and 35	1 (100)	0 (0)
HPV 16 and 56	2 (100)	0 (0)
HPV 31	0 (0)	1 (100)
HPV 35	3 (50)	3 (50)
HPV 66	0 (0)	1 (100)
HPV 16 and 31	0 (0)	1 (100)

HPV: human papilloma virus PD-L1: PD: programmed death-ligand

## Discussion

The human papillomavirus that is responsible for cervical carcinoma is a sexually transmitted infection. Persistent infection of the epithelial cells in the cervix leads to neoplastic transformation [20,21]. Studies utilizing cervical smears have recorded high-risk HPV prevalence in Nigeria to be between 16.6-25% [8,22,23]. In this study we detected high-risk HPV only in 51% of cervical carcinomas which is comparable to a study from Uganda that had HPV detection rate of 61% [24] as well as a study from Northern Nigeria that had a detection rate of 69.8% [25]. This is far cry from the HPV detection rate of 92.6% in cervical carcinoma by Omoseebi *et al*. in southwest Nigeria [11] and that of Takayanagi *et al*. who in their study in Japan had high-risk HPV detection rate of 91.5% [26] or that of Han *et al*. China with HPV detection rate of 92.7% [27]. Dom-Chima *et al*. [13] using next generation sequencing analysed 90 samples and discovered 44 HPV genotypes while type specific PCR could only detect 25 types as NGS was more sensitive but less specific. So, the cervical carcinomas in this study could be possibly caused by high-risk HPV not detected by type specific PCR. The study by Dom-Chima also did reveal only 10% of the identified genotypes were amongst the most prevalent [13]. The non detection of HPV DNA can also be as a result of extremely low number of the DNA copies to an extent below the threshold of sensitivity for detection [28]. The majority of cervical cancers, accounting for 82-88.7%, are caused by mono-infection, which is consistent with the findings of this study of 80.4% [10,27,28].

This was markedly different from another study from southwestern Nigeria that showed that mixed or multiple infections were the most common representing 68% of the cases [11]. The most common genotype involved in carcinoma is HPV16 either as a mono-infection or mixed with other genotypes (HPV18, 35, 56, 58 and 66) in this study; this is similar to studies by Sara da Mata *et al*. [10] and Prétet *et al*. [9]. In the study by Jean-Luc HPV 16 and 18 co-infection occurred in 10% of the cases and was the most predominant mixed-infection pairing which is similar to our study. All the multiple infections had HPV 16 as one of the genotypes in this study which is similar in trends to other studies that had HPV16 as present in 90 to 99% of cases of multiple infections [9-11,29]. The second most common HPV seen in this study is HPV 35 representing 17.6% of the cases which is similar to the study of Omoseebi *et al*. that had HPV 35 as the second most common genotype and represented 11.8% of the cases [11]. These findings are different from some other studies that recognized HPV 18 as the second most common genotype such as the multicentre study in France and a single centre Russian study [9,28]. A study from Martinque showed that rare genotypes of HPV51, and 68 were the most common [30].

Human papillomavirus positive tumours are associated with a higher number of tumour infiltrating lymphocytes [31]. In cervical cancer patients, there's an increased expression of immune response inhibitor receptors like PD-1 on CD3 cells and other immune cells. This allows neoplastic cells to evade the immune system through the PD-L1/PD-1 pathway [21]. This process applies a break on the body defence system in respect to the transformed cell thus avoiding elimination by the body´s immune system. The PD-L1/PD-1 has been showed to play a role in cervical carcinoma evolution, and it serves as target for immunotherapy [32]. There was no correlation between PD-L1 status and HPV status of cervical carcinoma in this study. Wessely *et al*. in a study of anal squamous cell carcinoma did not also demonstrate any correlation between HPV infection status and PD-L1 expression [31]. Conversely, Mezache *et al*. has showed that PD-L1 had increased expression in the presence of HPV-infected cells in the cervix [33]. Tang *et al*. also showed that in oral squamous cell carcinoma PD-L1 was overexpressed in HPV positive cases [34]. The variation in DNA copy numbers in cervical carcinoma can make the correlation of HPV status with PD-L1 expression status difficult [33].

The study is limited by the relatively small size of cases that were available for the study, this problem could have been eliminated with a prospective study, this reduces the power and generalizability of the study. The use of archival FFPE material is also a limitation, this is because poor storage can affect the quality of DNA available during extraction. The HPV detection kit used in this study is designed to detect mainly popular high-risk HPV and some rare high risk could be missed and this will result in a lower percentage of HPV associated cervical cancers. Also, this study used “laboratory only” PD-L1 clone which is different from the FDA approved clones for clinical application. Despite these limitations this study provides data on the prevailing genotypes in cervical carcinomas and the interaction with PD-L1.

## Conclusion

The most common HPV genotypes in our cohort is type 16 and 35. This is extremely important as the current vaccines in the country do not cover the range of HPV genotypes in our environment. The bivalent vaccine covers for type 16 and 18 while the nonvalent covers for types 6, 11, 16, 18, 31, 33, 45, 52, and 58. High risk HPV is detected in a significant number of cervical carcinoma cases and are majorly single infections. There is no correlation of PD-L1 expression and HPV infection status. Also, HPV infection status is not correlated with age or histological type or grade of cervical cancer.

### What is known about this topic

- *Human papillomavirus is the major cause of cervical cancer and previous studies have demonstrated high frequency of high-risk HPV associated cervical cancer;*
- *Programmed death ligand-1 immune pathway has been demonstrated to be very important in the pathogenesis of cervical cancer;*
- *The potentials of immunotherapy in the management of advanced cervical cancer*.

### What this study adds

- *The relatively common HPV 35 strain in invasive cervical cancer while noting that this genotype is not covered by the available vaccines;*
- *We did not find a significant relationship between human papillomavirus infection and PD-L1 expression in this cohort;*
- *Cervical cancer cells, including those affected by human papillomavirus, may express PD-L1 as a means of avoiding immune surveillance*.
